# Effects of Transcutaneous Electrical Nerve Stimulation on Pain and Chemotherapy-Induced Peripheral Neuropathy in Cancer Patients: A Systematic Review

**DOI:** 10.3390/medicina58020284

**Published:** 2022-02-14

**Authors:** Mirosława Püsküllüoğlu, Krzysztof A. Tomaszewski, Aleksandra Grela-Wojewoda, Renata Pacholczak-Madej, Florian Ebner

**Affiliations:** 1 Department of Clinical Oncology, Maria Sklodowska-Curie National Research Institute of Oncology, Krakow Branch, 00-001 Krakow, Poland; aleksandra.grela-wojewoda@onkologia.krakow.pl (A.G.-W.); renata.pacholczak@uj.edu.pl (R.P.-M.); 2 Labcorp (Polska) Sp. z o.o., 05-825 Warsaw, Poland; 3 Faculty of Medicine and Health Sciences, Andrzej Frycz Modrzewski Kraków University, 30-705 Kraków, Poland; krtomaszewski@gmail.com; 4 Scanmed St. Raphael Hospital, 30-693 Krakow, Poland; 5 Department of Anatomy, Medical College, Jagiellonian University, 31-008 Krakow, Poland; 6 Helios Amper-Klinikum Dachau, Akademisches Lehrkrankenhaus der Ludwig-Maximilians-Universität, 85221 München, Germany; Dr.Ebner@web.de

**Keywords:** cancer, pain, chemotherapy-induced peripheral neuropathy, cancer-related symptoms, pain management, patient-reported outcomes, physical therapy, transcutaneous electrical nerve stimulation, systematic review

## Abstract

Transcutaneous electrical nerve stimulation (TENS) is the usage of a mild electrical current through electrodes that stimulate nerves. Patients with malignancies experience pain and chemotherapy-induced peripheral neuropathy. A systematic review was performed to find research evaluating the effect of TENS on these two common symptoms decreasing the quality of life in cancer patients. PubMed, the Cochrane Central Register of Controlled Trials and EMBASE were searched. Original studies, namely randomized controlled trials, quasi-randomized controlled trials and controlled clinical trials, published between April 2007 and May 2020, were considered. The quality of the selected studies was assessed. Seven papers were incorporated in a qualitative synthesis, with 260 patients in total. The studies varied in terms of design, populations, endpoints, quality, treatment duration, procedures and follow-up period. Based on the results, no strict recommendations concerning TENS usage in the cancer patient population could be issued. However, the existing evidence allows us to state that TENS is a safe procedure that may be self-administered by the patients with malignancy in an attempt to relieve different types of pain. There is a need for multi-center, randomized clinical trials with a good methodological design and adequate sample size.

## 1. Introduction

Around 70% of cancer patients experience pain. It significantly influences health-related quality of life (HRQoL), being a subject of interest with regard to numerous instruments used for HRQoL assessment [1]. In half of cancer patients, pain is not adequately controlled [1]. Pain in patients with malignancies can be a result of the presence of the tumor mass (cancer-related pain), but it can also be related to treatment (e.g., radiotherapy) or postoperative and phantom pain. It is estimated that up to 40% of cancer survivors experience pain, which is treated with a multimodal approach. The main form of management remains medication, with oral being the preferable route. However, due to the development of non-pharmacological modalities, a combination of different techniques is currently applied [2,3,4,5,6]. Chemotherapy-induced peripheral neuropathy (CIPN) is a side effect, with sensory symptoms being pain, tingling, allodynia or numbness. Thus far, there has been no effective treatment for this condition; however, different techniques are being investigated [7,8,9,10,11]. CIPN is dose-limiting toxicity experienced by up to 40% of patients who receive neurotoxic chemotherapy. The most common drugs associated with this side effect are platins, vinca alcaloids, taxans, eribulin, bortezomib and thalidomide [7,10].

Transcutaneous electrical nerve stimulation (TENS) is the usage of a mild electrical current through electrodes that stimulate the nerves. The battery-powered hand-held stimulating device passes an electrical current through the intact skin surface. The TENS device modulates the frequency, width, duration and intensity of the pulse [12,13,14]. Johnson divided TENS into three main categories. The conventional TENS is defined as high frequency > 50 Hz and low intensity, which means causing paraesthesia without pain or motor contraction [12]. As the results of TENS are usually short-lasting but prompt in onset, the patients can self-administer the impulses as needed. TENS has numerous advantages: it is inexpensive, easy to use and implement, has no risk of overdose and entails reasonably few contraindications and side effects [15,16]. The procedure has certain limitations; generally, it should not be applied in areas with injuries, wounds or allodynia [15].

The reasons for TENS application are various [5]. In the population of patients with cancer, TENS has been mainly used for pain control purposes. TENS has also been commonly used for CIPN, fatigue, nausea and vomiting, constipation and xerostomia [14,16,17,18,19].

**Figure 1:**
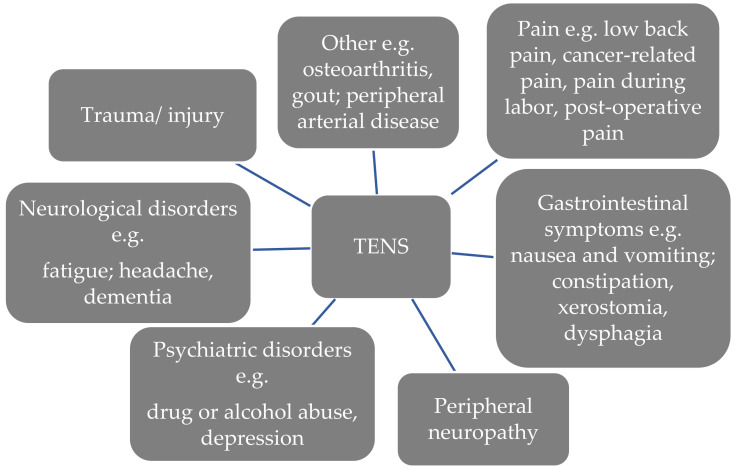
Reasons for TENS application in all patient groups [14,20].

Figure 1 presents examples of TENS usage reasons among all patient groups [14,20].

Thus far, two systematic reviews (SR) have been performed on TENS usage for cancer-related pain, the last one ten years ago. Both studies were inconclusive due to the insufficient amount of randomized controlled trials (RCTs) that could be included in the review [16,21]. The goal of the above-mentioned studies was to assess the effectiveness of TENS for cancer-related pain, while the goal of this study is to assess TENS’ effectiveness in cancer patients. In 2019, a Cochrane systematic review concerning TENS usage for chronic pain was published; however, it was not aimed at the cancer patient population [22]. Another systematic review concerning interventions in patients with dry mouth included cancer patients, among others [23]. In 2006, Ezzo et al. checked the influence of TENS on cancer-related nausea and vomiting, but later the review was withdrawn [24,25]. No systematic review concerning the effectiveness of TENS on CIPN treatment in cancer patients has been performed. However, a paper devoted to diabetic patients with neuropathy was published, with the conclusion that TENS can play a role as an effective tool in pain relief [26]. 

A review regarding neuromuscular electrical stimulation (NMES) usage and safety in cancer patients was performed. Nevertheless, due to critical differences between the two techniques, the conclusions cannot be transferred [27].

The aim of the current study was to assess whether TENS is effective for pain or chemotherapy-induced peripheral neuropathy management in comparison to sham TENS or no treatment or standard management in adult cancer patients.

## 2. Methods

The preferred reporting items for systematic reviews and meta-analyses (PRISMA) guidelines were used to perform a systematic literature search and review [28]. The main databases were searched: MEDLINE/ PubMed, Cochrane Central Register of Controlled Trials (CENTRAL)/ Willey Online Library and EMBASE/ OVID. Studies published up to May 2020 were included. The systematic review was registered online in the international prospective register for systematic reviews (PROSPERO) (No. CRD42020142014). The PRISMA checklist for this review is provided in the Supplementary Materials (Table S1) [29].

**Figure 2:**
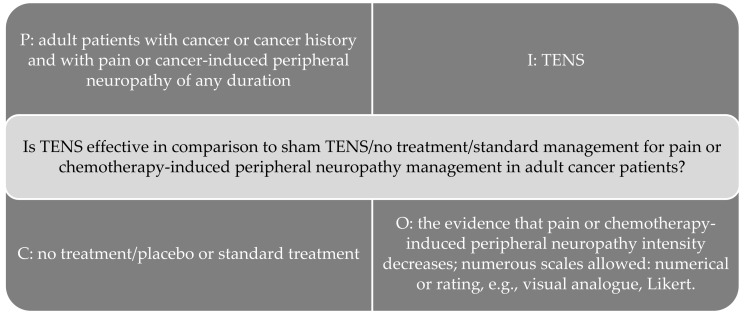
PICO strategy to build study question [30].

The study question was defined using the ‘patient, intervention, comparison, outcome’ (PICO) strategy, as shown in Figure 2 [30]. 

The MeSH terms used for the MEDLINE/PubMed search and the strategy applied for EMBASE are provided in the Supplementary Materials (Table S2). 

A systematic search was performed independently by MP and KAT in April 2020, and repeated by MP, KAT, AGW and RPM in August 2021. All titles and abstracts were revised by all researchers in order to check against the eligibility criteria. Chosen papers underwent full-text review, with reference screening for eligible papers. Any disagreement was discussed between the reviewers and a supervisor, FE, until agreement was achieved. 

The selection criteria are presented in Table 1. Insufficient data for analysis; unknown type of electrostimulation; and acupuncture-like stimulation or percutaneous or microcurrent electrical neuromuscular stimulation excluded the study from the analysis.

Additional quality assessment was performed with the usage of the validated five-point Oxford Quality Scale for included articles [31].

Data extracted from selected articles included the name of the first author; year of publication; patients’ data (number of patients included in the study, age); type of intervention, type of comparator intervention, and type of study/trial design; outcome of intervention (baseline and end of study outcomes); type of scale used to measure pain/CIPN intensity; quality of life data; scale used for quality of life data assessment; adverse events (AEs); intake of analgesics. Availability of data for result analysis was checked.

Outcome measures were as follows:

1. Primary—patient-reported pain/CIPN with the usage of commonly accepted and validated scales;
2. Secondary—patients’ functioning; quality of life; intake of painkillers; adverse events (patients’ safety);
3. Two authors, MA and KAT, judged the risk of the following biases in selected articles—selection bias; performance bias; detection bias; attrition bias; reporting bias. The Cochrane Collaboration’s risk of bias tool as described in the Cochrane Handbook for Systematic Review of Interventions was used. Any disagreement was resolved by a discussion between authors until a consensus was reached.

## 3. Results

### 3.1. Study Selection

**Figure 3:**
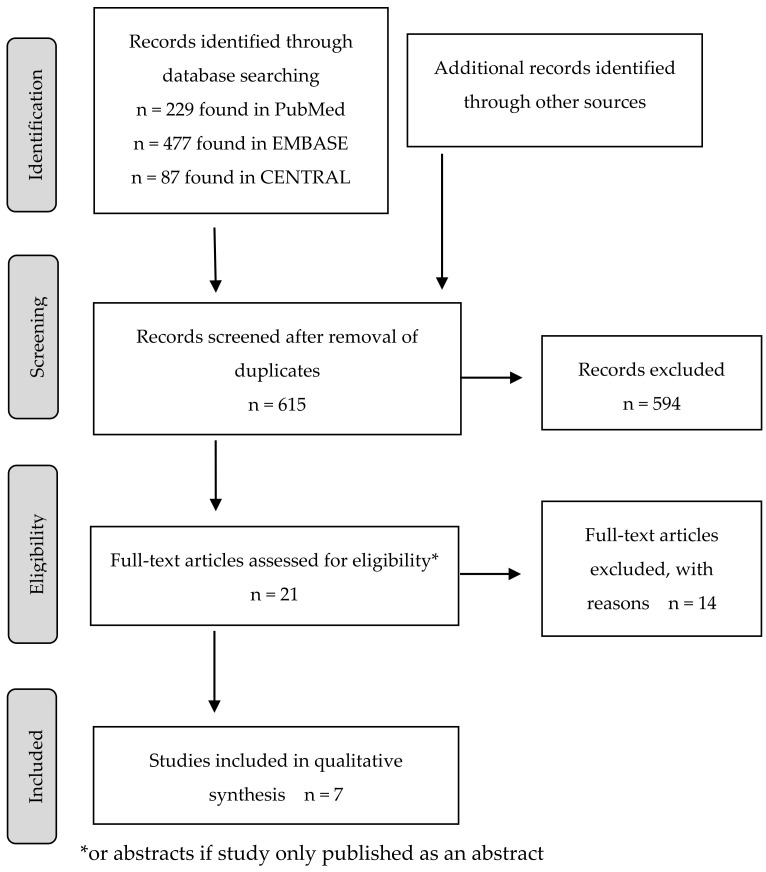
Study selection process.

The PRISMA flow chart of the study selection process is provided in Figure 3 [28]. After the database search, 793 articles were selected, including 229 from PubMed, 477 from EMBASE, and 87 from CENTRAL. Additionally, four articles were identified after citation screening, including Gadsby et al., which was used in Cochrane SR by Hurlow et al., but not included in this SR, as well as Erden and Celic, which was incorporated into this SR [16,32,33,34]. The removal of duplicates allowed the collection of 612 items. The titles and abstracts of these records were reviewed against the inclusion and exclusion criteria. This enabled the selection of 21 articles that underwent full-text assessment (if the full text was available) for eligibility. Of these, 14 were rejected with reason (mainly as follows: only abstract publication with insufficient amount of data available; no conventional TENS used as a study intervention; a comparator that was not a standard pain treatment or a study population that included patients without malignancy or under the age of 18), and seven studies were incorporated into a qualitative synthesis for this SR. The Rayyan program was used to store the hits and communicate between researchers.

### 3.2. Study Characteristics

Seven studies published between 2007 and 2020 were included in this SR [33,35,36,37,38,39,40]. A summary of the findings is shown in Table 2.

#### 3.2.1. Type of Studies Included

The study by Robb et al., (2007) was a randomized, placebo-controlled, blinded, crossover study. Bennett et al., (2010) performed a randomized, placebo-controlled, blinded, crossover feasibility study. Papers by Ferreira et al., 2011 and Fiorelli et al., (2012) described randomized, placebo-controlled trials. Erden and Celic (2015) performed a randomized controlled study. Lee et al., (2019) was a randomized, double-blinded, placebo-controlled, crossover pilot trial. Finally, Siemens et al., (2020) designed a randomized, blinded, sham-controlled, pilot, crossover study.

#### 3.2.2. Patient Population

All studies allowed only adult patients and included 260 patients in total (range between 24 and 50 participants) aged 18 to 79 years.

A description of the study populations is included in Table 2.

#### 3.2.3. Details of Pain Treated by Interventions

Details of pain managed by the intervention are collected in Table 2. In Robb et al., (2007), no criteria for pain intensity were specified. Analgesics usage was permitted. In the studies by Bennett et al., (2010), Lee et al., (2019), and Siemens et al., (2020), analgesic drugs intake was also allowed. Erden and Celic (2015) and Fiorelli et al., (2012) excluded patients with baseline pain. 

#### 3.2.4. Characteristics of Interventions

In all trials, the active arm involved conventional TENS (with high frequency of minimum 50 Hz and intensity causing paraesthesia without pain or motor contraction).

Detailed characteristics of interventions are presented in Table 2.

#### 3.2.5. Outcomes

1. Primary outcome (patient-reported pain/CIPN with the usage of commonly accepted and validated scales):

In Bennett et al., (2010), NRS and a visual rating scale (VRS) were used to measure pain intensity. The points were baseline, +30 min, and +60 min. Additionally, the Short-Form McGill Pain Questionnaire (SF-MPQ) was applied before and after the procedure to measure pain quality. The primary endpoints were pain intensity at rest and on movement at +60 min. 

To measure pain sensation, Robb et al., (2007) used the Brief Pain Inventory (BPI) short form, completed at baseline and then every week and after the end of the treatment: +3 months, +6 months, and +12 months. Pain relief was also measured in patients’ daily diaries.

Ferreira et al., (2011) used a visual analog scale (VAS) to measure pain intensity and severity at three time points: before the intervention, immediately after the intervention, and + 60 min. It was measured with patients at rest, with upper limb elevation, with a change in decubitus, and while coughing.

Fiorelli and colleagues (2012) reported pain by VAS before thoracotomy and then at +6 h, +12 h, +24 h, +48 h, +72 h, +96 h, and +120 h. 

Erden and Celic (2015) assessed pain with the usage of VAS at rest and while coughing at +24 h, +48 h, +96 h, and +120 h after the operation. 

In the study by Lee et al., (2019), the SF-MPQ and VAS (resting and function) were applied to measure overall pain and pain intensity before the procedure and during three visits thereafter. 

Siemens et al., (2020) used BPI with NRS to measure the change in pain intensity during the preceding 24 h as the primary outcome. To measure the change in pain perception during TENS, a 7-point VRS was applied.

2. Secondary outcomes (patients’ functioning; quality of life; intake of painkillers; adverse events):

In Bennett et al., (2010), the authors used their own patient satisfaction questionnaire. AEs were assessed according to common terminology criteria for adverse events (CTCAE) v 3.0 at the end of TENS application and 48 h after completing each procedure (via phone call).

Robb et al., (2007) applied the Hospital Anxiety and Depression Scale (HADS), which was completed at baseline and then every week and after the end of the treatment: +3 months, +6 months, and +12 months. Patients’ functioning (the movement of ipsilateral shoulder joint by the measurement of the degrees of the flexion and abduction) was assessed at baseline and at the end of the intervention (each time). Patients reported the usage of painkillers in their diaries. Additionally, the authors devised a questionnaire that was used after completion of a 12-week study to measure patients’ satisfaction with each intervention. AEs were monitored.

Fiorelli and colleagues (2012) reported respiratory function +72 h, +96 h, and +120 h after thoracotomy; additionally, the intake of narcotic medications received during the first 5 days after the operation was monitored.

Erden and Celic (2015) assessed analgesics usage +24 h, +48 h, +96 h, and +120 h after thoracotomy. 

In the study by Lee et al., (2019), the functioning (including speaking, tongue movement, and mouth opening) and fatigue intensity (with the usage of VAS) were measured before the procedure and during three visits thereafter. 

To assess HRQoL, the European Organization for Research and Treatment of Cancer quality of life questionnaire C30 was used in the study by Siemens et al., (2020). The analgesics intake was also documented in this study.

#### 3.2.6. Effects of Intervention

In the study by Robb et al., (2007), there were no statistically significant differences between groups in terms of worst pain, least pain, or average pain scores, as well as for pain relief scores from the patients’ pain diaries or BPI. Moreover, results for anxiety, depression, or the movement of the ipsilateral shoulder joint showed no differences between study arms. The authors reported that TENS ensured lower maximum pain interference scores (Wilcoxon *p* = 0.04, which was considered significant), although there were no differences when the least and last recorded interference scores were compared. When assessing the brief questionnaire, TENS was recorded to be significantly more effective than during other arms of the study (Chi-squared *p* < 0.001). Overall, 63% of participants that finished the study decided to continue using the device; the group was the largest for TENS (51%, *n* = 13, in comparison with 33%, *n* = 6 in placebo arm). Patients found the device easy to use and reported AEs to be minimal. The usage of painkillers was constant in 71% of patients (in all groups).

Bennett et al., 2010 suggest that TENS can improve cancer bone pain on movement, but not at rest. Overall, 63.2% of participants in the TENS group, in comparison to 26.3% in the placebo group, identified on the VRS at least good pain relief at +60 min. The difference in the proportion of patients experiencing at least good pain relief on movement with TENS compared to the placebo was statistically significant: 36.8% (95% CI (confidence interval) 7.55 to 66.2%). No statistical significance was reached when using the NRS score for pain intensity and pain relief on movement.

Overall, 37.5% of patients reported AEs (1.6 per patient); most of them were assessed as not related to TENS, with equal distribution in the active and placebo arms. Two withdrawals were connected to pain during the procedure (one during active TENS usage). Overall, the procedure was considered acceptable and safe.

The paper by Ferreira et al., (2011) showed that pain severity was significantly lower at rest in the active group immediately after TENS application (*p* = 0.038), and a trend was seen at +60 min with limb elevation (*p* = 0.05). The differences were not visible for coughing and changing decubitus. 

In the work by Fiorelli et al., (2012), pain assessed on the VAS was significantly lower in TENS than in the control group (*p* < 0.001) at different time points after the surgery: +6 h, +12 h, +24 h, +48 h, +96 h, and +120 h; moreover, pulmonary function was significantly better in the active arm. The morphine and non-opioid intake was lower in the TENS arm (*p* = 0.004 and *p* = 0.002, respectively). One participant from the active arm refused to continue with the trial; in another three cases (one from the active and two from the placebo arms), other factors did not allow the authors to collect all measurements from the patients.

In the study by Erden and Celic (2015), the TENS group scored significantly better in terms of pain level. At rest, the TENS group performed better at the time point of +72 h; during coughing, the active group had a lower pain level at +48 h, +72 h, and +96 h (*p* < 0.05). Additionally, the analgesics consumption intake was lower in the test group: for opioid consumption, it was statistically significant at +48 h, +72 h, and +96 h; for non-opioids, it was significant at +24 h (*p* < 0.05).

Lee et al., 2019 showed that resting pain measured by the SF-MPQ and VAS decreased more after TENS than placebo/no TENS and the results were statistically significant (*p* = 0.01 and *p* < 0.001, respectively, on both scales). There were no differences in terms of changes in pain with function and oral function. TENS was also statistically more effective in the treatment of fatigue in comparison to the no-TENS arm (*p* = 0.03)

In the study by Siemens et al., (2020), the change in pain intensity did not differ between groups. However, findings showed better responder rates for active TENS as a secondary outcome. One patient in each group found the procedure uncomfortable. The study showed TENS to be a safe and acceptable procedure, especially active in the case of women and people experiencing incidental pain. 

### 3.3. Risk of Bias

Risk of bias assessment with the usage of the Cochrane Collaboration’s risk of bias tool is presented in Table 3. Assessing the blinding of the TENS procedure seemed the most challenging part. The Oxford Quality Scale was incorporated to assess the RCTs’ quality [31]: Robb et al., (2007) scored four points, Bennett et al., (2010) also scored four points, Ferreira et al., (2011) received three points, Fiorelli et al., (2012) received five points and Erden and Celic (2015) only one point, Lee et al., (2019) scored four points and, finally, Siemens et al., (2020) also received four points.

## 4. Discussion

There is a very limited number of guidelines from international societies regarding cancer patients and TENS usage [41,42]. The recommendations from the American Cancer Society state that cancer-related pain can be alleviated by TENS [42].

In terms of safety, the work by Houghton and colleagues, which addresses the Guidelines of the Australian Physiotherapy Association and British Chartered Society of Physiotherapy, offers a chapter devoted to electrical stimulation and malignancy [43,44]. According to the authors, electrical stimulation should not be applied when malignancy is suspected or diagnosed, or in patients with pain and a history of malignancy within the previous five years. At the same time, it does not offer recommendations regarding TENS usage after this time period. TENS usage is only recommended for palliative pain management. One explanation is the possible stimulation of cancer dissemination by electrical stimulation. At the same time, the authors admit that the evidence for such conclusions is low. There are only preclinical data and assumptions that electrostimulation could promote deoxyribonucleic acid synthesis, cell replication, and act as a proangiogenic factor. Interestingly, the authors acknowledge that there are data supporting the possible anticancer activity of electrostimulation [43]. 

All trials selected for this SR revealed that TENS may show some effectiveness in certain areas of pain/CIPN treatment. However, due to the small sample sizes, it is difficult to draw any far-reaching conclusions, and there is a significant risk of producing incorrect estimates. The studies were not homogenous in terms of their design, population, comparison groups, outcomes, quality, treatment duration, procedure administration, or follow-up period. Additionally, studies lacked details allowing their replication.

Pain is a complex symptom, and, in these RCTs, it was assessed by both one-dimensional scales (such as VAS or NRS) and validated questionnaires. The number of instruments used in the included studies, as well as the different timing of pain measurement, makes it difficult to compare the results between the studies. Moreover, it was rarely described how patients were instructed about rating their pain severity on a chosen instrument. Of great value would be a simple statement indicating whether a measurement was performed during TENS or after its ending, as there are suggestions for maximum pain relief with ongoing use [45]. One may argue for the inclusion all types of pain in this SR. There are numerous reasons for pain in the cancer patient population and, in the majority of cases, individuals with malignancy experience more than one type of pain during the course of the disease and its treatment. Interestingly, there is no RCT devoted purely to TENS’ effectiveness in CIPN, although patients with this pain-related disorder could have been included in some of the studies described in this SR. A promising area for further exploration is acute postoperative pain in the lung cancer patient population. All included studies showed some effectiveness in pain treatment and two showed a reduction in analgesics consumption [33,36,37]. Although data are consistent with the results of a meta-analysis that examined postoperative settings in the general population, again, doubts about the quality of some studies, and the use of different time points and instruments for pain assessment, as well as setting different outcomes, make it impossible to draw conclusions [3].

Unlike in the study by Hurlow et al., (2012), the trial by Gadsby et al., (1997) was not included in this SR. The reason for this was the exclusion of studies using acupuncture-like TENS as a study intervention in this SR. There are data suggesting that some conditions, especially common in the cancer patient population (e.g., the usage of opioids), influence these two types of TENS (acupuncture-like and conventional) in a different manner. This statement makes reporting in trials on TENS conditions used as an intervention even more important.

There were very few AEs described in the studies, with no serious AEs, although only three of the trials reported them [35,37,39].

One study incorporated HRQoL as a secondary objective with the usage of a validated tool, and, therefore, it was impossible to report on this secondary outcome in this SR [40].

There was consistency with the results of other systematic reviews. Similarly to our study, the vast majority of SRs concerning the usage of TENS in various settings are inconclusive, e.g., concerning phantom pain, neuropathic pain, fibromyalgia, cancer-related pain, or chronic pain in adults [16,22,45,46,47]. However, there are some studies showing TENS’ advantage over a placebo, e.g., a meta-analysis of musculoskeletal pain [48]. All SRs reported the low quality of existing RCTs included in qualitative assessment and low confidence in the effect estimate stated. An issue with the quality of the studies assessing TENS’ effectiveness was described almost 10 years ago [49]. Non-optimal TENS dosing and inadequate outcome evaluation were the common flaws indicating low fidelity.

Economic analysis of TENS was not an aim of this study; however, an assessment of the cost-effectiveness of this procedure is being applied in planned RCTs (e.g., regarding the treatment of chronic dysmenorrhea [50]). Currently, the limited data do not support the superiority of TENS as an adjunct to primary care management of pain intensity in patients with tennis elbow in terms of cost-effectiveness [50]. However, some researchers indicate slightly lower costs of chronic back pain treatment in a group of patients receiving the TENS procedure [51]. 

This study has certain limitations. As TENS is commonly connected to acupuncture techniques, there are probably a number of studies performed in Asian countries, so involving a search of local databases for trials conducted in Asian countries could offer some new data. On the other hand, it is quite probable that searching the references for the chosen RCTs and existing related SRs would reveal such studies if they were of good quality. The authors of this SR decided not to follow this SR with a meta-analysis due to the limitations of the selected papers and did not contact the authors of the included RCTs if data were missing, in order to obtain a clearer picture of these studies. As the inclusion criteria allowed not only RCTs but also quasi-RCTs and controlled clinical trials, the study by Erden and Celic (2015) was not excluded from the qualitative analysis, even though it received only one point on the Jadad scale. The risk of missing articles in the literature search phase is a possible bias. We attempted to minimize this using a variety of adjusted key terms and repeating the process.

Following the results obtained in this research, we propose the following recommendations.

Recommendations for clinicians:

1. TENS cannot be implemented as a standard procedure for pain or CIPN treatment in the cancer patient population;
2. TENS can be used in patients with cancer and pain or CIPN, but this decision should be individualized and performed in a multidisciplinary team setting;
3. TENS is a safe procedure with a small number of AEs;
4. Staff should be trained in terms of what TENS is and where the cancer patients with pain or CIPN not reacting to standard treatment can access this treatment option.

Recommendations for patients:

1. Patients should be informed about the possibility of using TENS in the case of different types of pain or CIPN not responding to standard treatment;
2. Patients should be trained in terms of TENS device usage if they wish to use it;
3. Patients should not resign from the standard pain treatment in order to implement TENS;
4. If TENS application leads to the lowering of the dose of analgesics, it can be allowed;
5. TENS can be applied as a safe and easy-to-use procedure.

Recommendations for researchers planning future trials:

1. Validated scales (e.g., for pain measurement, HRQoL) should be implemented and patients should be trained in terms of using them;
2. Minimal sample size should be counted according to existing suggestions/power calculations should be applied;
3. Pain characteristics should be precisely described (pain at rest, on movement, relief in pain, maximal pain intensity, etc.);
4. Efforts should be applied to blind a procedure, patients, and staff;
5. Short- and long-term AEs should be followed up;
6. Follow-up period should be planned;
7. TENS settings should be described (e.g., type of device, frequency, intensity, length and frequency of application);
8. Planned trials should be multicenter;
9. Separate trials regarding the treatment of CIPN should be planned in a cancer patient population.

## 5. Conclusions

Currently, the data do not support the recommendation of TENS as a standard procedure for the treatment of different types of pain or CIPN in the cancer patient population. However, the existing evidence allows us to state that TENS is an easy-to-use and safe procedure that may be self-administered by patients with malignancy in an attempt to relieve different types of pain after individual consultation with healthcare specialists. 

To give clear instructions about the type of pain or/and specific cancer patient populations where TENS could be especially effective, future, well-designed trials with a longer follow-up period are needed. The existing data suggest pain relief on movement and acute postoperative pain as promising fields of study. Additionally, the sample size has been calculated and suggested for future trials [52,53]. 

## Figures and Tables

**Table 1:** Inclusion criteria used for study selection.

Topic	Study Inclusion Criteria
Population	Adult patients Conformation of malignancy, history of malignancy Confirmed pain or CIPN requiring treatment
Intervention	Conventional TENS applied in the area of pain or proximal to the pain over the nerve bundles
Comparator	Sham TENS No treatment Standard management for pain or CIPN
Outcome	Pain or CIPN intensity or duration measurement with the usage of approved/standard scales
Publication methodology	Randomized controlled trials Quasi-randomized controlled trials Controlled clinical trials
Publication type	Article or abstract of original study
Publication period	Studies published between April 2007 and May 2020
Language restrictions	Only English accepted

CIPN, chemotherapy-induced peripheral neuropathy; TENS, transcutaneous electrical nerve stimulation.

**Table 2:** Summary of the included studies.

	Robb et al.	Bennett et al.	Erden and Celic	Fiorelli et al.	Ferreira et al.	Lee et al.	Siemens et al.
Year of publication	2007	2010	2015	2012	2011	2019	2020
Year of conducting the study	UNK	UNK	2013	2008–2010	UNK	2011–2012	2016–2018
Country	United States	United Kingdom	Turkey	Italy	Brazil	United States	Germany
Patient population	49	24	40	50	30	41	26
Age	38–60	UNK^1^	24–76	UNK1	18–60	45–79	UNK ^1^
Comparator arm	TSE/placebo	Placebo TENS	No treatment	Placebo TENS	Placebo TENS	Placebo TENS/no treatment	Placebo TENS
Description of intervention	12 weeks, with 3 weeks for each type of intervention and 3 weeks of breaks in between (6 arms in total)	2 × 60 min (placebo and active TENS) with 2–7 days between treatments	Between 24th and 72nd hour after thoracotomy. TENS applied 3 times per day for 30 min, then twice daily till the removal of thoracotomy tube	First 5 days after thoracotomy. For the first 48 h every 4 h for 30 min, later twice daily	A one-time procedure in both arms. One day after the operation, one hour after epidural solution application	Three times for 40–45 min during radiotherapy treatment weeks 4th to 6th	Each procedure for 24 h with 24 h wash-out period in between; continuation of procedure as per patient choice
Type of population	Breast cancer female; without active malignancy	With any active malignancy; estimated survival > 4 weeks; with bone pain	Undergoing radical thoracotomy due to lung cancer	Undergoing radical thoracotomy due to lung cancer	Undergoing thoracotomy due to lung cancer	With H&N malignancy; during radiotherapy	With any type of cancer; receiving palliative support; estimated survival > 1 week
Crossover	Yes	Yes	No	No	No	Yes	Yes
Blinding	Yes	Yes	No	Yes	No	Yes	Yes
Drop-outs reported	Yes	Yes	No	Yes	No	Yes	Yes
Primary measure (for pain)	Pain report, pain relief, pain interference, anxiety and depression, arm mobility, and analgesic consumption. Time points: 3, 6, and 12 months	Pain intensity at rest and on movement at +60 min	Mean pain levels during rest and coughing at time points of 24 h, 48 h, 72 h, 96 h, 120 h	Pain change assessed on VAS at time points after the surgery	Pain change on VAS after TENS and +1 h, at rest, with change in decubitus moving the upper limbs, and coughing.	Overall pain and pain intensity (reported 30 min after procedure)	Change in pain intensity during the preceding 24 h
Main outcomes	No differences for worst pain, least pain, or average pain, pain relief scores from the patients’ pain diaries or BPI. For brief questionnaire, TENS significantly more effective than other arms	The difference in the proportion of patients experiencing at least good pain relief on movement with TENS compared to placebo was statistically significant. No significance was seen when using NRS score for pain intensity and pain relief on movement	TENS group scored significantly better for pain level. At rest, TENS group performed better at time point of +72 h; during coughing, the active group had lower pain level at +48 h, +72 h, and +96 h	Pain assessed on VAS was significantly lower in TENS than in the control group at several time points after the surgery: +6 h, +12 h, +24 h, +48 h, +96 h, and +120 h	Pain severity was significantly lower at rest in active group immediately after TENS application	Resting pain measured by SF-MPQ and VAS decreased more after TENS than placebo/no TENS and the results were statistically significant	Change in pain intensity did not differ between groups. Better responder rates for active TENS as a secondary outcome
Type of pain measured	Chronic of min. 6-month duration, treatment-related	Caused by bone metastases from any malignancy. Intensity min. 3/10 NRS	Acute and related to surgical procedure	Acute and related to surgical procedure	Acute and related to surgical procedure	Pain during radiotherapy with intensity of min. 1/10 on NRS	Any, cancer- or treatment-related pain with intensity of min. 3/10 on NRS
Scale used for pain assessment	BPI Patients’ diaries A brief questionnaire	VRS, NRS SF-MPQ	VAS	VAS	VAS	VAS SF-MPQ	BPI NRS VRS
CIPN allowed	Yes	UNK (no allodynia)	NA	NA	NA	UNK	Yes
Function assessment	Yes	No	No	Yes	No	Yes	No
AEs assessment	Yes	Yes	No	No	No	No	Yes
HRQoL assessment	Yes	No	No	No	No	No	Yes
Patients’ satisfaction assessment	Yes	Yes	No	No	No	No	No
Analgesics intake evaluation	Yes	No	Yes	Yes	No	No	Yes
Follow-up	12 months	1 h, then 48 h	120 h	120 h	60 min	UNK	24 h, then flexible

^1^ Mean age provided separately for each study arm; AE, adverse event; BPI, Brief Pain Inventory; CIPN, chemotherapy-induced peripheral neuropathy; HRQoL, health-related quality of life; NA, not applicable; NRS, numerical rating scale; SF-MPQ, Short-Form McGill Pain Questionnaire; TENS, transcutaneous electrical nerve stimulation; TSE, transcutaneous spinal electroanalgesia; UNK, unknown; VAS, visual analog scale; VRS, visual rating scale.

**Table 3:** Risk of bias assessment.

	Robb et al.,2007	Bennett et al.,2010	Erden and Celic 2015	Fiorelli et al., 2012	Ferreira et al., 2011	Lee et al., 2019	Siemens et al., 2020
Random sequence generation (selection bias)	+	+	+	+	+	+	+
Allocation concealment (selection bias)	?	+	−	+	?	?	+
Blinding participants and personnel (performance bias)	−	−	−	+	−	+	+
Blinding outcome assessment (detection bias)	?	?	−	+	?	?	?
Incomplete outcome data (attrition bias)	?	+	−	+	−	−	−
Selective reporting (reporting bias)	+	+	+	+	+	+	+

## Supplementary Materials

The following are available online at https://www.mdpi.com/article/10.3390/medicina58020284/s1, Table S1: PRISMA checklist for the review, Table S2: Literature review search terms.
