# Supplementary material for: Effects of Transcutaneous Electrical Nerve Stimulation on Pain and Chemotherapy-Induced Peripheral Neuropathy in Cancer Patients: A Systematic Review
Source: Medicina (Kaunas). 2022 Feb 14;58(2):284. doi: 10.3390/medicina58020284 (PMC8876365; doi:10.3390/medicina58020284)
Supplement: Supplementary file 1 [file medicina-58-00284-s001.zip › Table S2.pdf]

Table S2. Literature review search terms.

Database	Search terms
MEDLINE/PubMed	"Transcutaneous electrical nerve stimulation"[All Fields] OR (transcutaneous[All Fields] AND electrical[All Fields] AND nerve[All Fields] AND stimulation[All Fields]) OR electrostimulation[All Fields] OR TENS[All Fields]<br>AND cancer[Title/Abstract] OR cancers[Title/Abstract] OR carcinoma[Title/Abstract] OR carcinomas[Title/Abstract] OR tumor[Title/Abstract] OR tumors[Title/Abstract] OR tumour[Title/Abstract] OR tumours[Title/Abstract] OR oncology[Title/Abstract] OR oncological[Title/Abstract] OR malignant[Title/Abstract] OR malignancy[Title/Abstract] OR malignancies[Title/Abstract] OR neoplasm[Title/Abstract]) OR neoplasms[Title/Abstract] OR neoplastic[Title/Abstract] AND pain[Title/Abstract) OR neuropathy[Title/Abstract]
EMBASE*	1. exp Transcutaneous electrical nerve stimulation/<br>2. (transcutaneous and electrical and nerve and stimulation).mp.<br>3. electrostimulation.mp.<br>4. TENS.mp.<br>5. 1 or 2 or 3 or 4<br>6. (cancer* or carcinoma* or tumor* or tumour* or oncolog* or malignan* or neoplas* or carcinom*).ti,ab<br>7. pain*.ti,ab<br>8. neuropath*.ti,ab<br>9. 7 or 8<br>10. 5 and 6 and 9

* For EMBASE (and accordingly for Cochrane Central Register of Controlled Trials) the search strategy was modified (e.g. "All Fields" were replaced by ".mp")
